# Status Set Sequential Pattern Mining Considering Time Windows and Periodic Analysis of Patterns

**DOI:** 10.3390/e23060738

**Published:** 2021-06-11

**Authors:** Shenghan Zhou, Houxiang Liu, Bang Chen, Wenkui Hou, Xinpeng Ji, Yue Zhang, Wenbing Chang, Yiyong Xiao

**Affiliations:** School of Reliability and Systems Engineering, Beihang University, Beijing 100191, China; zhoush@buaa.edu.cn (S.Z.); zy1914125@buaa.edu.cn (H.L.); bang@buaa.edu.cn (B.C.); houwk@buaa.edu.cn (W.H.); sy1914103@buaa.edu.cn (X.J.); Zhangyue1127@buaa.edu.cn (Y.Z.); changwenbing@buaa.edu.cn (W.C.)

**Keywords:** data mining, status set sequential pattern mining, time window, TW-Apriori algorithm, periodicity analysis

## Abstract

The traditional sequential pattern mining method is carried out considering the whole time period and often ignores the sequential patterns that only occur in local time windows, as well as possible periodicity. Therefore, in order to overcome the limitations of traditional methods, this paper proposes status set sequential pattern mining with time windows (SSPMTW). In contrast to traditional methods, the item status is considered, and time windows, minimum confidence, minimum coverage, minimum factor set ratios and other constraints are added to mine more valuable rules in local time windows. The periodicity of these rules is also analyzed. According to the proposed method, this paper improves the Apriori algorithm, proposes the TW-Apriori algorithm, and explains the basic idea of the algorithm. Then, the feasibility, validity and efficiency of the proposed method and algorithm are verified by small-scale and large-scale examples. In a large-scale numerical example solution, the influence of various constraints on the mining results is analyzed. Finally, the solution results of SSPM and SSPMTW are compared and analyzed, and it is suggested that SSPMTW can excavate the laws existing in local time windows and analyze the periodicity of the laws, which solves the problem of SSPM ignoring the laws existing in local time windows and overcomes the limitations of traditional sequential pattern mining algorithms. In addition, the rules mined by SSPMTW reduce the entropy of the system.

## 1. Introduction

Data mining is a research field that has gained increasing attention in recent years, and sequential pattern mining is an important subtopic of data mining research. However, the traditional sequential pattern mining method has the following limitations: the traditional method is to mine the database considering the whole time period, which may ignore the sequential patterns that only occur in the local time period. For example, in hot summers, heat stroke is a common disease, and severe heat stroke often produces hyponatremia, heat stroke and other complications. However, there is no causal relationship between the disease and its complications, but a correlation relationship; thus, the sequence pattern of “heatstroke → complications” is often difficult to uncover when looking at the whole study period, but it is easy to find these rules when summer or even a hot period is selected as the study time window. In addition, traditional sequential pattern mining only considers the ID of the item, which cannot find the relationship between items with a different status. Traditional methods only consider the support constraint, which may lead to the mining of sequential patterns with low regularity and practical value.

In order to solve the above problems, this paper proposes the status set sequential pattern mining method with time windows (SSPMTW). The constraints of the time window, minimum confidence, minimum coverage and minimum factor set ratio are introduced to mine the sequential patterns in the local time window, and the periodic analysis of these sequential patterns is carried out so that the mined rules are the focus of users and have more regularity and practical value. In addition, SSPMTW considers the status of the items so that sequential patterns with status attributes can be mined. Aiming at the method proposed in this paper, this paper improved the Apriori algorithm, and proposes a TW-Apriori algorithm so as to complete the status set sequential pattern mining with time window and analyze the sequential pattern periodically.

### 1.1. Related Work

#### 1.1.1. Research on Sequence Pattern Mining Method

Scholars across the world have conducted in-depth research on sequence pattern mining methods. This paper will describe the research status of sequence pattern mining methods from the following four aspects.

##### Sequential Pattern Mining with Time Windows

Sequential pattern mining with time windows is rarely studied, but association rule mining with time windows has been studied by some researchers. Xiao et al. [1] studied association rule mining with time windows in real transaction databases and proposed a TW-Apriori algorithm for frequent itemset mining with time windows. Xiao et al. [2] proposed a variable neighborhood search algorithm (VNS) for mining frequent itemsets with maximum time windows. Xiao et al. [3] proposed the VNS algorithm to optimize the maximum frequent time window selection problem in the sequence database of association rules. Zabihi et al. [4] proposed a novel fuzzy sequential pattern algorithm with a sliding window constraint which permits elements of a pattern to span a set of transactions within a user-specified window. Therefore, loss of useful sequences is prevented in the search process.

##### Periodic Sequence Pattern Mining

Periodic association rules were first proposed by Ozden et al. [5], who studied the association rules with periodicity that appear repeatedly in the database. Li and Jitender [6] proposed a method of mining partial periodic association rules in a time series database, which can mine association rules over a periodic period of time without restricting them to a fixed rule and sequence. Manziba [7] proposed a periodic sequence pattern mining algorithm, which does not rely on the user to obtain periodic correlation values but can also mine all types of periodic patterns at the same time. Han et al. [8] has studied some periodic patterns in time series database and proposed relevant algorithms to mine them efficiently. Based on the temporal Apriori algorithm, Li and Ning et al. [9] mined association rules with year, month and day as a cycle. Yang et al. [10] believed that the interference of random noise may lead to the asynchrony of a periodic mode, so he put forward the asynchronous periodic mode model. Huang and Chang [11] studied mining asynchronous short period patterns in temporal database, and each time point contained multiple events. Lee and Jiang et al. [12] relaxed the periodicity to a fuzzy periodicity and proposed an algorithm to mine the fuzzy periodicity association rule.

##### Study on Constraints of Sequential Pattern Mining

The constraints of sequential pattern mining will affect the number and type of frequent itemsets and sequential patterns. How to set the constraints and thresholds reasonably is a key problem. In order to solve the problem that a constant support threshold may cause long itemsets and sequences to be ignored, Seno and Karypis [13] combined LPMiner and SLPMER algorithms to mine long frequent itemsets and frequent sequence patterns. When the length of frequent itemsets and frequent sequences is longer, the support threshold is smaller. Wangand Karypis [14] proposed a BAMBOO algorithm to mine closed itemsets based on decreasing support. Yun and Leggett [15] proposed a WLPMiner algorithm to construct a constraint that decreases in support as the length increases.

Yun [16] proposed a weighted and interesting sequential pattern mining method based on similarity support or weight level. Yun and Leggett [17] proposed a weighted frequent pattern mining method whose support decreases with increasing sequence pattern length. Chang [18] proposed a weighted sequential pattern mining method that considers time intervals in sequential databases.

Massegliaet al. [19] raised the issue of incremental mining of sequence patterns. When new transactions or customers join the original database, the cost of frequent sequence pattern mining from an updated database is reduced by using previously mined useful information. Ng et al. [20] proposed a sequence pattern mining method that takes into account time intervals so as to predict the time interval for any two transactions of a frequent sequence.

##### Closed Sequence Pattern Mining

A closed sequence pattern refers to the absence of any other contained sequence pattern under the same support. It not only fully expresses the complete set of results but also has a more streamlined result without information decay. In order to solve the problem that the traditional closed sequential pattern mining algorithm will produce a large number of inefficient redundant patterns when the support threshold is low and the sequential patterns in the database are diverse, Jingsong Zhang et al. [21] proposed the CCSpan algorithm. Fabregue et al. [22] proposed the OrderSpan algorithm, which extracts a closed set of partially ordered patterns from a sequence database. Moreover, the OrderSpan is extended by using efficient optimization methods used in the field of sequential pattern mining.

#### 1.1.2. Research on Sequence Pattern Mining Algorithm

R. Agrawal and R. Srikant [23] proposed an algorithm based on the Apriori feature, which considers that all non-empty subsequences of the sequence pattern are sequence patterns. Based on this property, some Apriori-like algorithms have been proposed successively, which mainly contain two mining ideas: (1) horizontal data format mining ideas: the AprioiAll, AprioriSome, and DynamicSome algorithms proposed by R. Srikant and R. Agrawal [24] and the GSP algorithm proposed by F. Masseglia et al. [25]; (2) vertical data format mining ideas, such as the classical SPADE algorithm proposed by M. Zaki [26] and the SPAM algorithm proposed by Ayres et al. [27].

In addition, Yu et al. [28] used a Boolean matrix to improve the Apriori algorithm and implemented the algorithm on the Hadoop platform. Youcefet al. [29] proposed the GA Apriori and PSO Apriori algorithm by combining a genetic algorithm and particle swarm optimization algorithm. Du [30] proposed an improved Apriori algorithm based on a Boolean matrix and sort index rules. Anil vasoya et al. [31] improved the Apriori algorithm by combining distributed computing with parallel computing so as to find frequent itemsets from large databases in a shorter time. Agustinet al. [32] proposed COP algorithm based on the PrefixSpan algorithm to optimize the correlation of results obtained in the process of sequential pattern mining.

### 1.2. The Main Contribution of This Paper

At present, there is little research on status set sequential pattern mining with time windows, but this has great research value and significance in practical application. It can discover easily ignored rules in local windows and mine the periodicity of these rules.

The traditional sequential pattern mining methods only consider the ID of items in the temporal database, which ignores the relationship between items with different status. Additionally, the traditional sequential pattern mining methods only have the support constraint, which may lead to the mining rules still lacking application value. More importantly, the traditional sequential pattern mining methods are to mine the temporal database over the whole time period and count the itemsets and sequences, while the sequential patterns existing in the local event window are often ignored. To solve the above problems, this paper improved the traditional sequential pattern mining methods.

Considering the status attribute of each item in the database for some complex systems and considering the status of the item, we can find the system between the normal state and the fault state at different time points, and then, according to the information mined, we can carry out preventive maintenance for the system that is about to have a fault state in advance.

The time window, confidence degree, coverage, factor set ratio and other constraints are introduced to mine the sequence patterns existing in local time windows, to find out the more regular sequence patterns among them and to mine the factor sets that lead to the occurrence of some key itemsets in the local time window.

The periodicity of the sequential pattern with the time window is analyzed. The law of periodic occurrence often has higher application value.

Through the improvement of traditional methods, this paper proposes the status set sequential pattern mining method with time window; it also improved the Apriori algorithm and proposes the TW-Apriori algorithm to mine the status set sequential pattern with time window.

### 1.3. The Main Content of This Paper

In the Section 2, the related concepts and constraints of status set sequential pattern mining with time windows are explained; in the Section 3, the main ideas of the TW-Apriori algorithm proposed in this paper are explained, and the algorithm is verified by a small-scale example; in the Section 4, the algorithm is verified by a large-scale example, and the results are analyzed; in the Section 5, the conclusion of this paper is given.

## 2. Problem Description for SSPMTW

### 2.1. Related Definitions and Properties

The following defines important concepts in the mining process of status set sequence patterns with time windows, where Table 1 is the symbol definition of the status set sequence pattern mining model with time windows.

**Definition 1.**  **Status Itemset (SI):** *An itemset can be defined as a status itemset if and only if it meets the following constraints:*

1. X⊆I;
2. $\forall i\in X,\;i\in \left\{1,2\right\}$;
3. ∀j∈I−X, j=0.

**Definition 2.**  **Frequent Status Itemset with Time Windows (FSITW):** *FSITW can be expressed as*$X^{W}$*if and only if it meets the following constraints:*

1. X⊆I;
2. $\forall w_{i},w_{j}\in W,\;w_{i}\;⋂\;w_{j}=\emptyset$;
3. $\forall w\epsilon W,\;\frac{\left|D{\left(X\right)}^{w}\right|}{\left|D^{w}\right|}\times 100\%\geq s\%$.

When mining frequent status itemsets with time windows, this paper draws on the downward closure property of the Apriori algorithm, puts forward the two concepts of subset and superset and proposes three new important properties based on these to calculate efficiency. This is shown below.

**Definition 3.**  **Subset:** *The set of status items within*$\left(t_{s},t_{e}\right)$*the time window can be represented as*$X^{\left(t_{s},t_{e}\right)}$*, if*$X^{\prime \left(t_{s}^{\prime },t_{e}^{\prime }\right)}$*is a subset of*$X^{\left(t_{s},t_{e}\right)}$*, if and only if*$X^{\prime }\subseteq X,t_{s}^{\prime }<t_{e}^{\prime },t_{s}^{\prime }\geq t_{s},t_{e}^{\prime }\leq t_{e}$.

**Definition 4.**  **Superset:***The set of status items within*$\left(t_{s},t_{e}\right)$*the time window can be represented as*$X^{\left(t_{s},t_{e}\right)}$*, if*$X^{\prime \left(t_{s}^{\prime },t_{e}^{\prime }\right)}$*is a superset of*$X^{\left(t_{s},t_{e}\right)}$*, if and only if*$X\subseteq X^{\prime },t_{s}^{\prime }<t_{e}^{\prime },t_{s}^{\prime }\leq t_{s},t_{e}^{\prime }\geq t_{e}$.

**Property 1.**  *If status itemset*$X\left(X\subseteq I\right)$*is frequent in the time window*$\left(t_{s},t_{e}\right)$*, then any subset of it must also be frequent in the time window*$\left(t_{s},t_{e}\right)$.

**Proof.**  Since the status itemset $X\left(X\subseteq I\right)$ is frequent in the time window $\left(t_{s},t_{e}\right)$, $\left|D{\left(X\right)}^{\left(t_{s},t_{e}\right)}\right|/\left|D^{\left(t_{s},t_{e}\right)}\right|\times 100\%\geq s\%$. Since $X^{\prime }$ is a subset of $X,\;\left|D{\left(X^{\prime }\right)}^{\left(t_{s},t_{e}\right)}\right|\geq \left|D{\left(X\right)}^{\left(t_{s},t_{e}\right)}\right|$, $\left|D{\left(X^{\prime }\right)}^{\left(t_{s},t_{e}\right)}\right|/\left|D^{\left(t_{s},t_{e}\right)}\right|\times 100\%\geq s\%$. Thus, $X^{\prime }$ is also frequent in the time window $\left(t_{s},t_{e}\right)$. □

**Property 2:**  *If status itemset*$X\left(X\subseteq I\right)$*is infrequent in the time window*$\left(t_{s},t_{e}\right)$*, then any superset of it must also be infrequent in the time window*$\left(t_{s},t_{e}\right)$.

**Proof.**  Since the status itemset $X\left(X\subseteq I\right)$ is infrequent in the time window $\left(t_{s},t_{e}\right)$, $\left|D{\left(X\right)}^{\left(t_{s},t_{e}\right)}\right|/\left|D^{\left(t_{s},t_{e}\right)}\right|\times 100\%\leq s\%$. Since $X^{\prime }$ is a superset of X, $\left|D{\left(X^{\prime }\right)}^{\left(t_{s},t_{e}\right)}\right|\leq \left|D{\left(X\right)}^{\left(t_{s},t_{e}\right)}\right|$, $\left|D{\left(X^{\prime }\right)}^{\left(t_{s},t_{e}\right)}\right|/\left|D^{\left(t_{s},t_{e}\right)}\right|\times 100\%\leq s\%$. Thus, $X^{\prime }$ is also infrequent in the time window $\left(t_{s},t_{e}\right)$. □

**Property 3.**  *If status itemset* $X\left(X\subseteq I\right)$ *is infrequent in the time window* $W_{1}$ *, status itemset* $Y\left(Y\subseteq I\right)$ *is infrequent on* $W_{2}$ *, and* $W_{1}⋂W_{2}=\emptyset ⋂W_{2}=\emptyset$ *, so the status itemset* Z=X⋃Y *must be infrequent in the time window* $W_{3}=W_{1}⋃W_{2}.$

**Proof.**  Since the status itemset $X\left(X\subseteq I\right)$ is infrequent in the time window $W_{1}$ and the status itemset Z=X⋃Y is a superset of X, according to property 2, Z is infrequent in the time window $W_{1}$. Similarly, Z is infrequent in the time window $W_{2}$, i.e., $\left|D{\left(Z\right)}^{W_{1}}\right|/\left|D^{W_{1}}\right|\times 100\%\leq s\%,\;\left|D{\left(Z\right)}^{W_{2}}\right|/\left|D^{W_{2}}\right|\times 100\%\leq s\%$. Assuming $\left|D^{W_{1}}\right|$ is greater than $\left|D^{W_{2}}\right|$, then $\left|D{\left(Z\right)}^{W_{3}}\right|/\left|D^{W_{3}}\right|\times 100\%\leq \left|D{\left(Z\right)}^{W_{1}}+D{\left(Z\right)}^{W_{2}}\right|/2\left|D^{W_{1}}\right|\times 100\%\leq s\%.$ Thus, the status itemset Z=X⋃Y is infrequent in the time window $W_{3}=W_{1}⋃W_{2}$. □

These three important properties have an important value and role in frequent status set mining and can greatly reduce the number of candidate status itemsets, thus greatly improving the computational efficiency.

**Definition 5.**  **Status-Set Sequence with Time Windows (SSTW):** *SSTW is a collection of orderedstatus items with time windows such as*$X\to^{W}Y$*,*X,Y⊆FSITW*, and W represents the time window in which*X→Y*occurs.*

There are also two important properties when searching for a candidate SSTW, which can greatly improve the speed of the algorithm, as shown in Properties 4 and 5 below.

**Property 4.**  *If the status set sequence*$SS\left(SS\subseteq I\right)$*in the time window*$\left(t_{s},t_{e}\right)$*is frequent, then any subset of its status set sequence must also be frequent in the time window*$\left(t_{s},t_{e}\right)$.

**Proof.**  Since the status set sequence $SS\left(SS\subseteq I\right)$ is frequent in the time window $\left(t_{s},t_{e}\right)$, ${\left|SS\right|}^{\left(t_{s},t_{e}\right)}/\left|D^{\left(t_{s},t_{e}\right)}\right|\times 100\%\geq s\%$. Since $SS^{\prime }$ is a subset of SS, ${\left|SS^{\prime }\right|}^{\left(t_{s},t_{e}\right)}\geq {\left|SS\right|}^{\left(t_{s},t_{e}\right)}$, ${\left|SS^{\prime }\right|}^{\left(t_{s},t_{e}\right)}/\left|D^{\left(t_{s},t_{e}\right)}\right|\times 100\%\geq s\%$. Thus, $SS^{\prime }$ is also frequent in the time window $\left(t_{s},t_{e}\right)$. □

**Property 5.**  *If the status set sequence*$SS\left(SS\subseteq I\right)$*in the time window*$\left(t_{s},t_{e}\right)$*is infrequent, then any of its super status set sequences must also be infrequent in the time window*$\left(t_{s},t_{e}\right)$.

**Proof.**  Because the status set sequence $SS\left(SS\subseteq I\right)$ is infrequent in the time window $\left(t_{s},t_{e}\right)$, ${\left|SS\right|}^{\left(t_{s},t_{e}\right)}/\left|D^{\left(t_{s},t_{e}\right)}\right|\times 100\%\leq s\%$. Since $SS^{\prime }$ is a superset of SS, ${\left|SS^{\prime }\right|}^{\left(t_{s},t_{e}\right)}\leq {\left|SS\right|}^{\left(t_{s},t_{e}\right)}$, ${\left|SS^{\prime }\right|}^{\left(t_{s},t_{e}\right)}/\left|D^{\left(t_{s},t_{e}\right)}\right|\times 100\%\leq s\%$. Thus, $SS^{\prime }$ is also infrequent in the time window $\left(t_{s},t_{e}\right)$. □

**Definition 6.**  **Mean Support of SSTW:** *The average support for SSTW of the form*$X\to^{W}Y$*can be defined as*$\bar{s}\%=\left(\sum_{w\in W}\left|w\right|\cdot s_{w}\right)/\left(\sum_{w\in W}\left|w\right|\right)\times 100\%$*, where*$s_{w}$*is the support of*$X\to^{W}Y$*, i.e.,*$s_{w}=\left(\left|D{\left(X\to Y\right)}^{w}\right|\right)/\left(\left|D^{w}\right|\right),\forall w\in W$.

**Definition 7.**  **Mean Confidence of SSTW:** *The average confidence for SSTW of the form*$X\to^{W}Y$*can be defined as*$\bar{c}\%=\left(\sum_{w\in W}\left|w\right|\cdot c_{w}\right)/\left(\sum_{w\in W}\left|w\right|\right)\times 100\%$*, where*$c_{w}$*is the confidence of*$X\to^{W}Y$*, i.e., *$c_{w}=\left(\left|D{\left(X\to Y\right)}^{w}\right|\right)/\left(\left|D{\left(X\right)}^{w}\right|\right),\forall w\in W$.

**Definition 8.**  **Time Coverage Rate of SSPTW:***The time coverage of*$X\to^{W}Y$*, tcr%, indicates the coverage of the SSPTW over the entire time period, which can be expressed as follows:*

(1) $$tcr\%=\frac{\sum_{w\in W}\left|w\right|}{\left|T\right|}\times 100\%$$

*where*$\sum_{w\in W}\left|w\right|$*is the total length of the time window in which*$X\to^{W}Y$*resides,*$\left|T\right|$*represents the total time interval of the time series database. In special cases, when*tcr%=100%*, the SSPTW is a traditional, full-time sequence pattern; when*tcr%≤100%*, the SSPTW is a part-time sequence pattern. Since the width of the minimum time window*ω*is set in this paper and the following research will also be based on the minimum time window,*tcr%*must satisfy the following restrictions:*tcr%≥ω/T.

**Definition 9.**  **Status Set Sequential Pattern with Time Windows (SSPTW):** $X\to^{W}Y$*is SSPTW, if and only if it meets the following constraints:*

1. X⊆SI, Y⊆SI, X⋂Y=∅, W≠∅;
2. $X^{W},Y^{W}\subseteq FSITWs$, that is, $X^{W}$ and $Y^{W}$ meet the user-defined minimum support s%;
3. ∀w∈W,$\;\left(\left|D{\left(X\to Y\right)}^{w}\right|\right)/\left(\left|D^{w}\right|\right)\times 100\%\geq s\%$;
4. ∀w∈W,$\;\left(\left|D{\left(X\to Y\right)}^{w}\right|\right)/\left(\left|D{\left(X\right)}^{w}\right|\right)\times 100\%\geq c\%$;
5. ∀w∈W,$\;\left|w\right|\geq \omega$.

Based on the above definition, it is known that an SSPTW’s average support is always not less than the minimum support, and its average confidence is not less than the minimum confidence; otherwise, a sequence with large average support and average confidence cannot ensure that it is an SSPTW.

**Definition 10.**  **Coverage Rate (CR) of SSPTW:** *The coverage of the status set sequence,*$SSPTW={\left\{X_{1}\to X_{2}\to_{\cdots }\to X_{k}\right\}}^{W}$*, can be expressed as:*

(2) $$CR\left(SSPTW\right)=min\left\{\frac{{\left|X_{1}\to X_{2}\to X_{3}\to_{\cdots }\to X_{k}\right|}^{W}}{{\left|X_{k}\right|}^{W}}\times 100\%|\forall k=2,3{,}_{\cdots },p\right\}$$

**Definition 11.**  **Strong Status Set Sequential Pattern with Time Windows (Strong SSPTW):** *The* *status set sequence pattern (SSPTW) is a strong* *status set sequence pattern if and only if* $CR\left(SSPTW\right)\geq mincov$ *, where* mincov *is the user-defined minimum coverage threshold (d%), which is assigned the same value as the minimum confidence threshold ( c%).*

**Definition 12.**  **Factor Set of FSI (FS):** *The factor set of status itemset*X*can be expressed as*$FS\left(X\right)$*, for any status set sequence pattern*ssp*in*$\left\{ssp_{1},ssp_{2}{,}_{\cdots },ssp_{i}\right\}$*; if*$ssp\to^{W}X$*is still a status set sequence pattern, then*ssp*is an element of*$FS\left(X\right)$*. The factor set of the frequent status itemset*X can be expressed as:

(3) $$Rate\left(X\right)=\underset{i}{\sum }\left(CR\left(ssp_{i}\to^{W}X\right)\right)$$

*The*$ssp_{i}$*is an element in factor set*$FS\left(X\right)$*, and*i is the subscript of each element.

*When it satisfies Formula (2.4), where*minfs*is the user-defined minimum factor set ratio*u%*,*$FS\left(X\right)$*is called the main factor set of frequent status set*X.

(4) $$Rate\left(X\right)=\underset{i}{\sum }\left(CR\left(ssp_{i}\to^{W}X\right)\right)\geq minfs$$

### 2.2. Periodic Analysis of SSPTW

A periodic status set sequence pattern is a collection of sequence patterns and a series of time windows, such as $X\to^{P_{i}}Y$, where $P_{i}$ is a periodic time window containing specific events. Key concepts in the mining process of periodic status set sequential patterns are defined below.

**Definition 13.**  **Periodic Width, T:***This is the user-specified width of a time window that satisfies the periodic pattern, where the periodic width is an integer multiple of the minimum time window, that is,*T=k×ω.

**Definition 14.**  **Periodic Interval, O:** *This is a user-defined time window interval that satisfies a periodic pattern, where the periodic interval is an integer multiple of the minimum time window, that is,* O=k×ω *, where the periodic interval can be determined by experience by day, week, month, year, etc.*

**Definition 15.**  **Periodic SSP:** *A status set sequence pattern, such as*X→Y*, when both the periodic width*T*and periodic interval*O*are satisfied. The status set sequence pattern is the periodic status set sequence pattern, which is expressed as*$X\to^{T,O}Y$.

**Definition 16.**  **Periodic Time Coverage Rate (PTCR):** *Assume that*p*is the number of cycles for a time window that satisfies a periodic SSP and n-p is the number of cycles for a time window that does not meet that periodic SSP.*$X\to^{T,O}Y$*is a periodic SSP with strong regularity if and only if*$X\to^{T,O}Y$*satisfies the following periodic time coverage values:*

(5) $$\frac{p}{n-p}\times 100\%\geq e\%$$

*Among them,* e% *is the user-defined minimum periodic time coverage threshold. When a periodic pattern* $X\to^{P_{i}}Y$ *satisfies both* T *and* O *constraint thresholds, but* $PTCR\left(X\to^{P_{i}}Y\right)<e\%$ *, this is a periodic SSP with weak periodic time coverage rules.*

**Definition 17.**  **Periodic Analysis of Patterns:** *Periodic analysis of patterns refers to the mining of repeated status set sequence patterns with periodic regularity in a time series database.*

**Figure 1:**
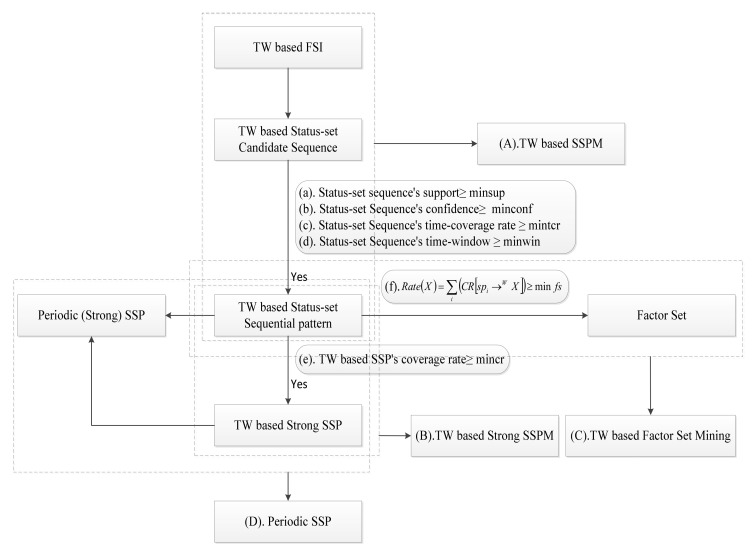
Frame diagram of SSPM with time window.

Figure 1 is a framework for SSPMTW. In this paper, the sequential pattern mining model is extended to mine SSP with time windows, strong SSP with time windows, the main factor set of a frequent status itemset with time windows and periodic sequential patterns.

## 3. SSPTW Mining Algorithm

### 3.1. Mining Large-One FSITWs

Mining the largest set of frequent one-status items with time windows (large-one FSITWs) are the most important part of status set sequence pattern mining with time windows because these are the basis for producing n-FSITWs and SSPTW. Since the time window of FSITW must be no less than the user-defined minimum time window threshold of minwin, a simple method of mining large-one FSITWs can take the following two steps:

(1) Divide the time series database into several time periods through minwin;
(2) Count the support degree of individual items in each time period of the database and compare with the user-defined minimum support degree to determine whether the set of status items are frequent. If the set of status items are frequent in one time window and not frequent in adjacent time windows, the two time windows are merged, and the support degree of the set of status items on the merged time window is counted. If the minimum support threshold is still met, the set of status items are considered frequent in the merged time window.

Based on this idea, this paper maximizes the time window of the frequent one-status itemset in preparation for finding frequent n-FSITWs in the next step. The time interval for frequent time windows obtained by this method will be an integer multiple of minwin.

### 3.2. Research on SSPMTW Mining Algorithm

The SSPTW mining steps are as follows:

(A) Mining a set of frequent status items with time windows, FSITW.
(B) Mining the status set sequence pattern with time windows, SSPTW.
(C) Mining periodic status set sequence patterns with time windows, periodic SSPTW.

#### 3.2.1. FSITW Algorithm

This paper proposes the TW-Apriori algorithm to mine SSPMTW and analyze its periodicity. The main idea of the algorithm is introduced below.

The traditional sequential pattern mining algorithm is to mine frequent itemsets in the whole temporal database, ignoring the frequent itemsets in the local time window. The frequent itemsets are the basis of mining sequential patterns, so the traditional sequential pattern mining algorithms cannot mine the sequential patterns in the local time window. Therefore, mining frequent status itemsets FSITWs in local time window is the key.

Generating large-one FSITWs is the basis of mining frequent status itemsets with time windows. This algorithm divides the database into small segments according to the minwin threshold, then scans the whole database, counts the support of one-candidate status itemsets in each small time segment, compares them with minsup to determine whether they are frequent, and uses the idea described in Section 3.1 to maximize the time window of one-candidate status itemsets by combining the adjacent time window. If the time coverage rate of the time window where the one-candidate status itemset is located meets mintcr, then the one-candidate status itemset is large-one FSITWs.

**Figure 2:**
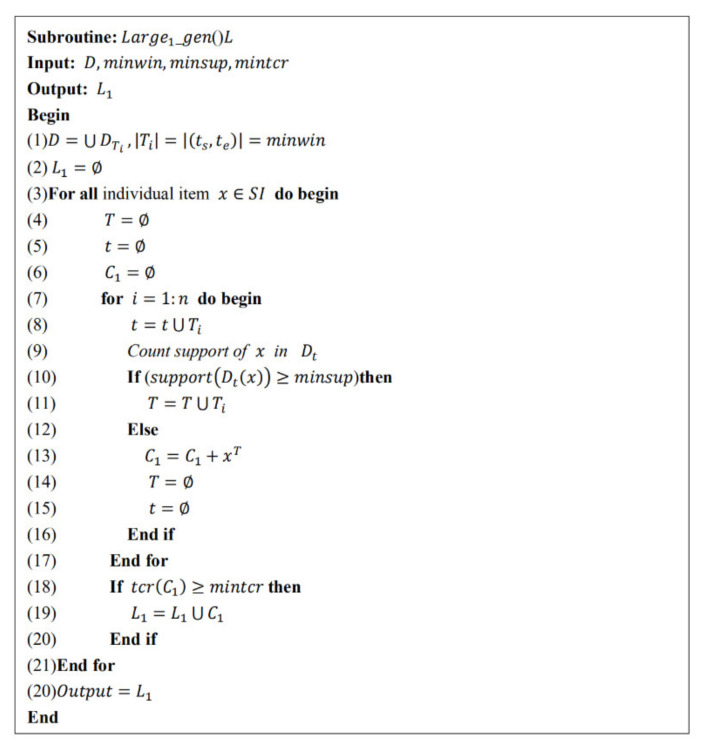
Large-one FSITWs mining algorithm.

The specific pseudo code is shown in Figure 2. The inputs are the temporal database, minwin, minsup and mintcr, and the outputs are large-one FSITWs.

When mining FSITWs, we need to generate candidate k-FSITWs (k≥2). The idea of the algorithm is to generate the next candidate k-FSITWs through the (k-1)-FSITWs mined in the previous step until no candidate status itemset is generated. It is worth mentioning that for all candidate status itemsets, only when their time window is in the intersection of their time window supersets can we count their support, which is also the core idea of property 3 in Section 2.

**Figure 3:**
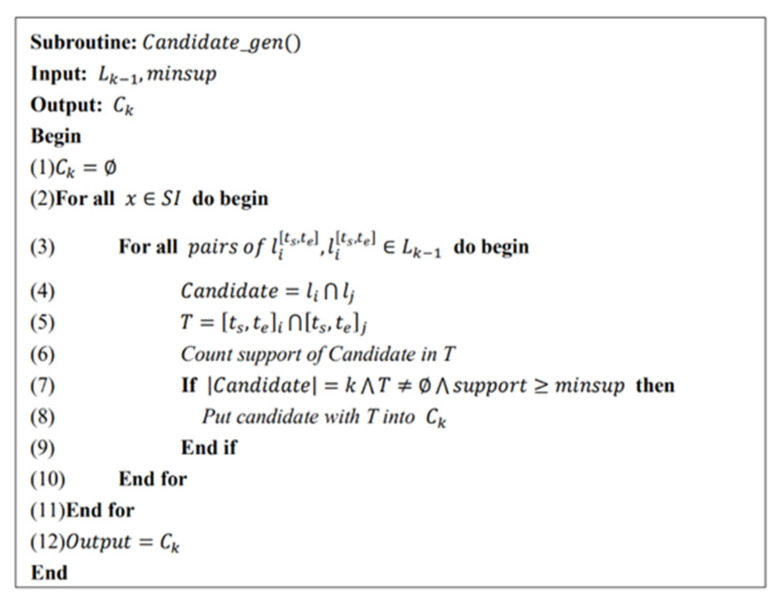
New candidate FSITWs mining algorithm.

The specific pseudo code is shown in Figure 3. The inputs are the (k-1)-FSITWs mined in the previous step and minsup, and the outputs are the candidate k-FSITWs.

**Figure 4:**
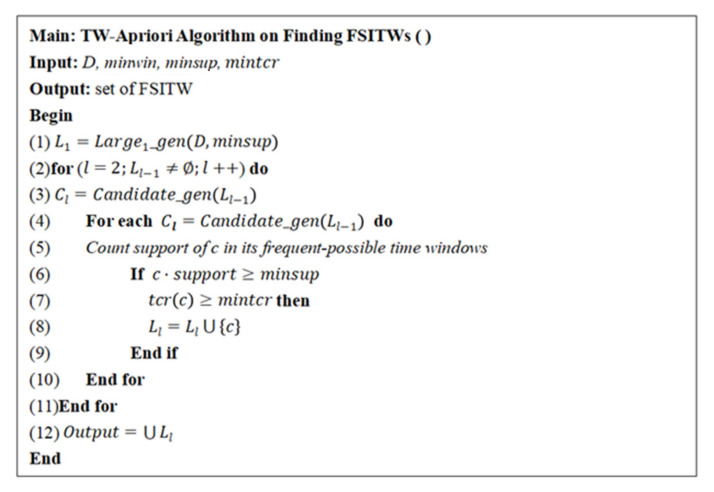
TW-Apriori algorithm for mining FSITWs.

Based on the above two subroutines, this paper provides the pseudo code for mining FSITWs, as shown in Figure 4. First, large-one FSITWs are generated, and then, all candidate k-FSITWs are generated by using the subroutine shown in Figure 3, and their support and time coverage rate are counted. When the minimum support threshold and minimum time coverage rate threshold are met, FSITWs can be obtained. In this paper, we will use the properties 1–3 proposed in the second section to reduce the generation of candidate itemsets so as to improve the efficiency of the algorithm.

Using the above three algorithms, we finally mined all the frequent status itemsets and their time windows to prepare for the next sequential pattern with time windows.

#### 3.2.2. SSPTW Algorithm

**Figure 5:**
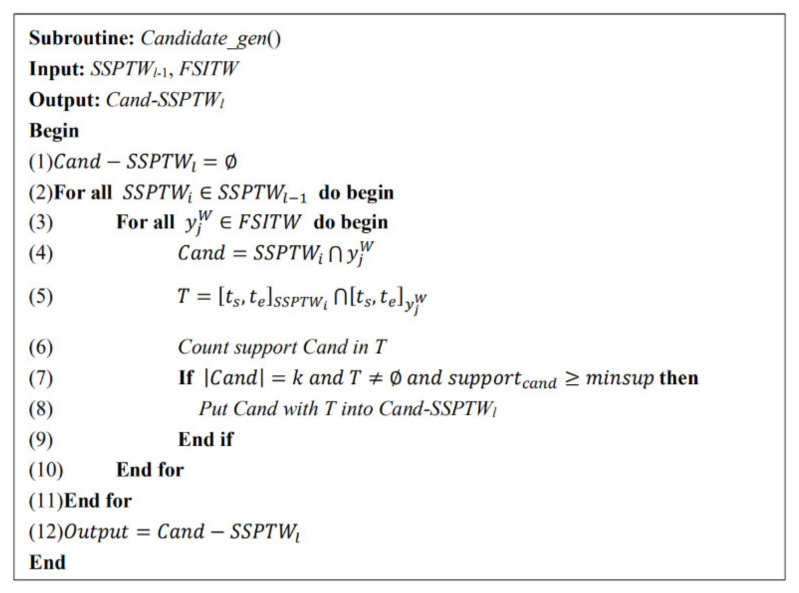
New Candidate-SSPTW mining algorithm.

All FSITWs have been mined in Section 3.3.1, which are the basis of mining status set sequential patterns with time windows. In the process of mining SSPTW, we need to combine (k-1)-SSPTW(k≥2) and 1-SSPTW to generate candidate k-SSPTW, in which 1-SSPTW is all FSITWs. The specific pseudo code is shown in Figure 5. Its inputs are the (k-1)-SSPTW mined in the previous step and FSITWs, and its outputs are the candidate k-SSPTW.

**Figure 6:**
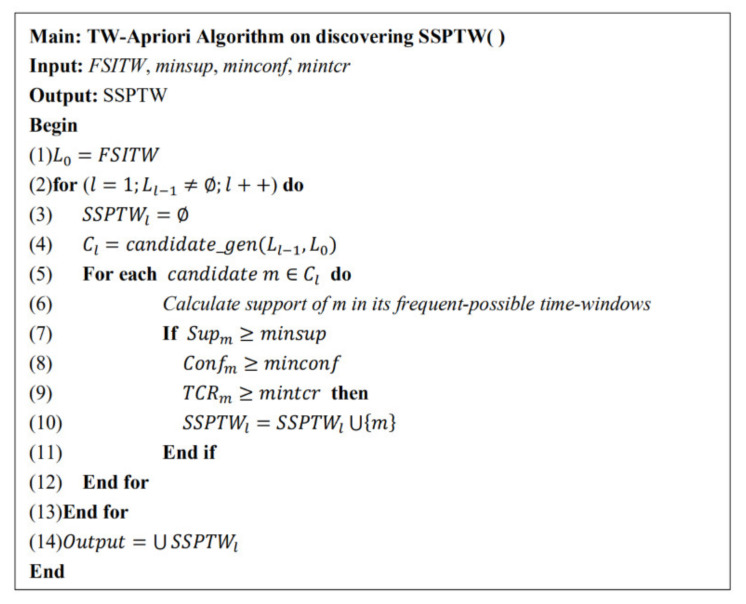
TW-Apriori algorithm for mining SSPTW.

Using the above subroutine, we can obtain all candidate SSPTWs, and calculate their support, confidence and time coverage rate so as to mine SSPTW. Figure 6 shows the specific pseudo code of the SSPTW mining algorithm.

#### 3.2.3. Strong SSPTW Algorithm

**Figure 7:**
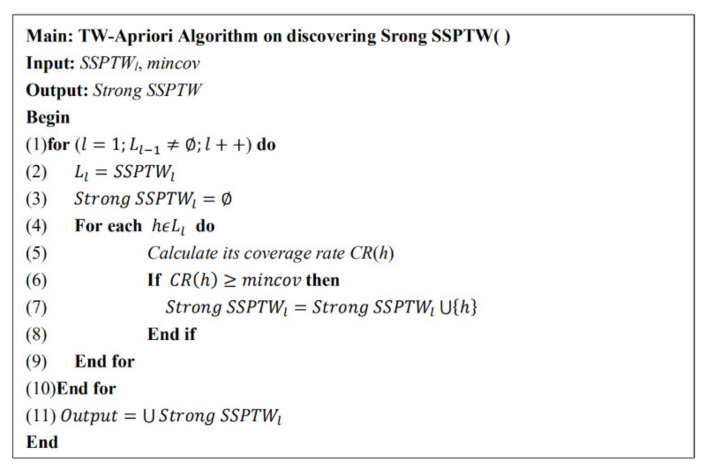
TW-Apriori algorithm for mining Strong SSP.

After the study in Section 3.2.2, all the status set sequence patterns with time windows will be finally discovered. In order to solve the problem that the sequence pattern mined by the traditional algorithm has weak regularity and low practical application value, this paper adds the constraint of coverage rate to mine SSPTW with stronger rules, that is, strong SSPTW. Figure 7 is the specific pseudo code of the strong SSPTW mining algorithm. By counting the coverage of SSPTW and judging whether it meets the coverage threshold, the strong SSPTW is mined.

#### 3.2.4. Factor Set of FSITW Algorithm

In the practical application process, users may be interested in some important FSITWs, because when these important FSITWs occur, they may bring huge economic benefits or huge losses, or other important impacts. Therefore, the constraint of the minimum factor set ratio is added in this paper.

**Figure 8:**
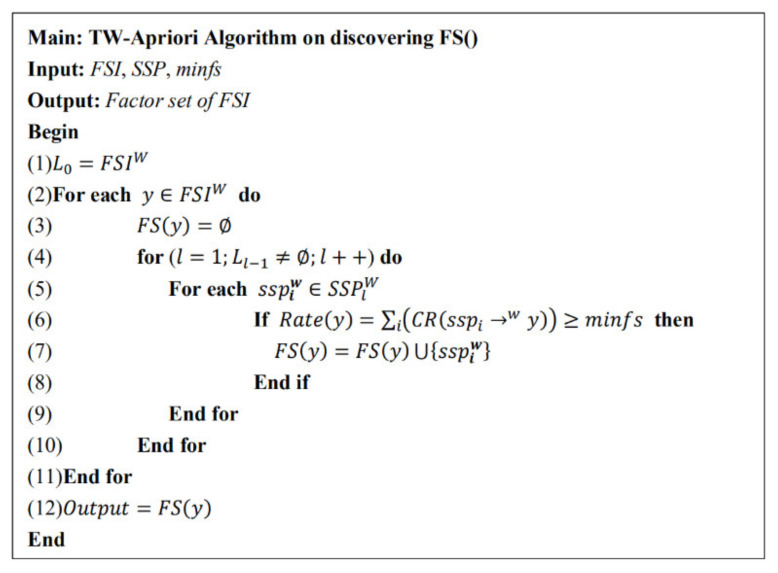
TW-Apriori algorithm for mining the FSI factor set.

Figure 8 shows the specific pseudo code of the factor set of the FSITWs mining algorithm. According to definition 12 in Section 2, when the factor set of the FSITWs meets the minimum factor set ratio, it is the main factor set of the FSITWs.

#### 3.2.5. Periodic SSP Algorithm

By analyzing the periodicity of the status set sequence pattern with time windows, the regularity of the periodicity in the pattern is found. Firstly, the SSPTW and its time windows satisfying the period width T and period interval O are discovered, and then, the periodic time coverage rate of these patterns is calculated. When they meet the minimum periodic coverage rate threshold, the pattern is determined as a periodic status set sequence pattern.

**Figure 9:**
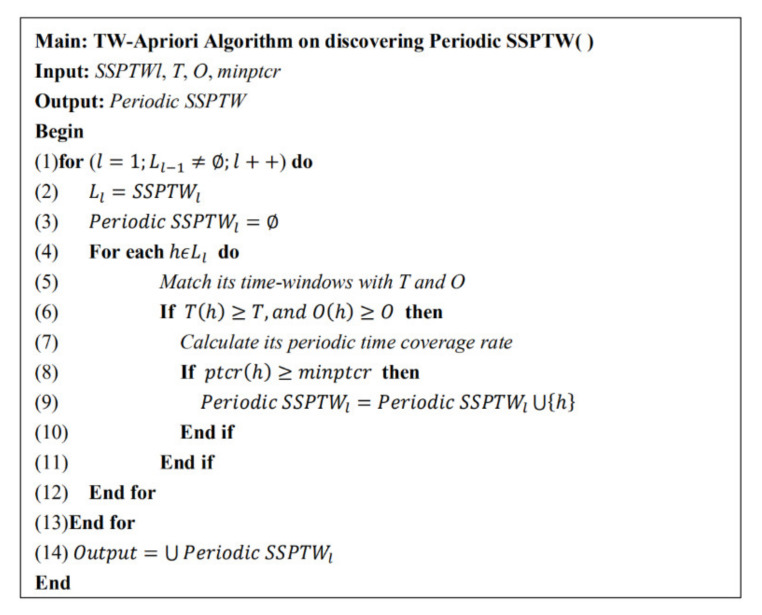
Periodic sequence pattern mining algorithm.

The specific pseudo code of the algorithm idea is shown in Figure 9.

#### 3.2.6. Analysis of Computational Complexity of the Algorithm

When mining frequent itemsets with time windows, candidate frequent itemsets are obtained by pairwise combination of the previous frequent itemsets; this is also the case for the mining of status sets sequential patterns with time windows. This will generate a large number of candidate itemsets and sequences. For example, when n (k-1)-FSITWs are mined in the previous step, $C_{n}^{2}$ candidate k-FSITWs will be generated. This will greatly occupy the memory of the computer and increase the computational complexity of the algorithm. The properties 1 to 5 proposed in the second section can greatly reduce the generation of candidate frequent itemsets and status sets sequential patterns, reducing the complexity and improving the efficiency of the algorithm.

#### 3.2.7. Entropy in the Systems

Entropy is ubiquitous in the system, and its physical meaning is a measure of the degree of system chaos. The method proposed in this paper is to mine the potential rules in the system, so as to predict the future state of the system. These rules are sequential patterns, which reflect the relationship between different system states. The appearance of one state of the system may cause the occurrence of another state. Through these sequential patterns, we can know the future state of the system, so as to optimize the system. Therefore, the discovery of these sequential patterns can make the system more orderly and stable, and correspondingly, the entropy of the system decreases.

### 3.3. Solution and Analysis of an Example of SSPMTW

The following is a small-scale example to verify the feasibility and validity of the methods and algorithms presented in this paper.

Table 2 shows 25 instances of fault monitoring for three parts of a system, recording the corresponding data in chronological order and finally forming the time series database. The TID represents the timestamp of the monitoring; the Status itemset represents the set of status items for each monitoring record; the three parts correspond to three items— $\left\{i_{1},i_{2},i_{3}\right\}$, $\mathrm{status}\in \left\{0,1,2\right\}$, 0—normal, 1—potential failure, 2—failure. minwin=5, minsup=40%, minconf=60%, mincov=60%, mintcr=40%, minfs=80%.

#### 3.3.1. Solution and Analysis of SSPMTW

##### Step 1. Mining FSITW

This section uses two methods to mine frequent status itemsets. The first method is FSI mining without considering time windows. The second method is mining frequent status itemsets with time windows, that is, the FSITW mining method in this section. Using two different solutions, the two methods are compared and analyzed.

First, in order to easily calculate the support of each item, the temporal database needs to be converted to a 0–1 form, as shown in Table 3.

**Figure 10:**
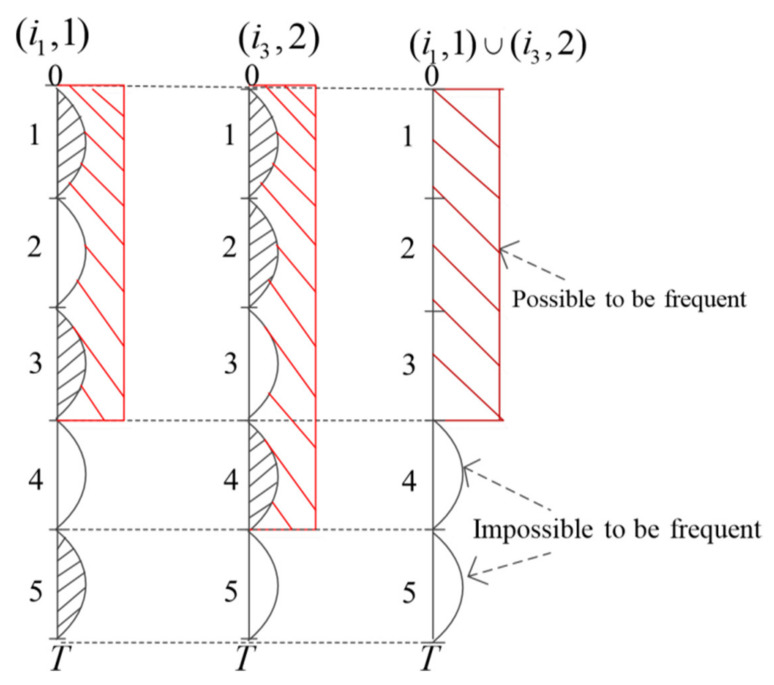
An example illustrating FSITW.

(1) FSI Mining
  FSI Mining takes place over the entire time period of the database. If the support of a status itemset is not less than the minimum support specified by the user, it is determined that the set is a frequent status itemset. By summing the columns of each status item in Table 3, the support of each status item is calculated (as shown in red in the last line of Table 3). Since misup = 40%, the minimum amount of support is 25*40% = 10, which means that when the amount of support for each item is not less than 10, the item is frequent. As can be seen from the red letters in the last row of Table 3, only (i_1_,2) of the status items meets the requirements, and all other items have less than 10 support, so the set of frequent status items finally mined by the FSI Mining method is {(i_1_,2)}.
(2) Frequent status Item Set Mining with Time Window
  Frequent status itemsets with time windows are divided into small time periods by minwin, and then, the support of itemsets is determined to be minsup-satisfied in a small time period. When minsup is satisfied, the time coverage of these time windows is determined to be no less than mintcr. Only when both conditions are satisfied can the itemset be determined to be FSITW.
  (A) 1-FSITW
    Figure 10 takes two status items, (i_1_,1) and (i_3_,2), as examples for analysis. The graphics show that (i_1_,1) is frequent in time windows [1,3,5] and infrequent in time windows [2,4]; (i_3_,2) is frequent in time windows [1,2,4] and infrequent in time windows [3,5].
    The time window maximization of frequent one-status itemsets means that, if the minsup constraint threshold is still satisfied after merging the frequent time window and the adjacent infrequent time window, we will merge their time windows.
    (i_1_,1) is frequent in time windows [1,3,5] but is not frequent in time windows [2,4]. However, by maximizing the time window of frequent one-status itemsets, we find that (i_1_,1) is also frequent in time windows [1–3], and its support is 6/15, which meets minsup. Then, by considering the minimum time coverage threshold, we find that tcr(i_1_,1) = 3/5 > 40%, so the maximum frequent time windows of (i_1_,1) are [1–3,5]. Similarly, using the same method, we find that the frequent time windows of (i_3_,1) are [1–4].
    Summarily, using the same method, we mined the frequent one-status itemsets with the largest time window, namely 1-MFSITW.
  (B) k-FSITW
    Based on the 1-FSITW, this paper finally mines all FSITWs, and makes a simple comparison between FSITW and FSI, as shown in Table 4.

It can be seen from the table that 10 FSITWs satisfying the conditions can be mined in the local time window, while only one FSI can be mined in the whole time period. Through comparison, it can be found that the proposed method can mine those status itemsets that are not frequent in the whole time period but are frequent in the local time window.

##### Step 2. Mining SSPTWs

When mining sequential patterns of status sets with time windows, we need to consider the constraints of support, confidence and time coverage. Only when the sequence meets the threshold of minsup, minconf and mintcr can it be judged as sequential patterns of status sets with time windows. Among them, minsup = 40%, minconf = 60%, mintcr = 40%. The specific SSPTW is shown in Table 5. In the following table, L_1_ represents an SSPTW with a length of 1, that is, all FSITWS, and L_K_ represents the k-status set sequence pattern with time window.

##### Step 3. Mining Strong SSPTWs

On the basis of SSPTWs, strong SSPTWs can be mined by considering the coverage threshold, where mincov = 60%.

It can be seen from the “coverage rate” column in Table 5 that all SSPTWs meet the mincov constraint, that is, SSPTWs in Table 5 are strong SSPTWs.

##### Step 4. Mining Factor Set of FSITWs

When considering the factor set rate constraint, we can mine the major factor set of FSITW with time windows. We took frequent status itemsets with time windows {(i_2_,2)-[1–3,5]} as an example, in which we assumed that (i_2_,2) was an important part that users pay close attention to. Finally, we discovered the major factor set of (i_2_,2), as shown in Table 6.

In Table 6, we can find all the major factor sets that may lead to (i_2_,2)–[1–3,5]. At the same time, we sorted the support, confidence and time coverage of each SSPTW that led to (i_2_,2)–[1–3,5] from large to small so that we could quickly lock high-frequency and effective SSPTWs, which is convenient for users to focus on monitoring these sequential patterns.

#### 3.3.2. Periodic Analysis of SSPTW

Using the periodic analysis of SSPTWs in Table 5, we finally found periodic SSPs, as shown in Table 7.

It can be seen from Table 7 that there are two periodic laws in the table: the periodic time windows are [1,3,5], [2,4] and the cycle width of these two windows is 1; the cycle interval is also 1, and the periodic time coverage is 100% and 66.7%, respectively.

Table 7 not only excavates periodic SSP but also sorts them according to support, confidence, coverage rate and TCR, so that users can select relatively high value periodic status set sequence patterns.

## 4. Large-Scale Example Experiment

This section will use large-scale examples to verify the feasibility and efficiency of the methods and algorithms proposed in the article. The data used in this section include 96,554 sets of transaction data composed of 1000 items. The description of the dataset is shown in Table 8.

### 4.1. Analysis of Solution Results

#### 4.1.1. TW-Apriori Algorithm Efficiency Verification

**Figure 11:**
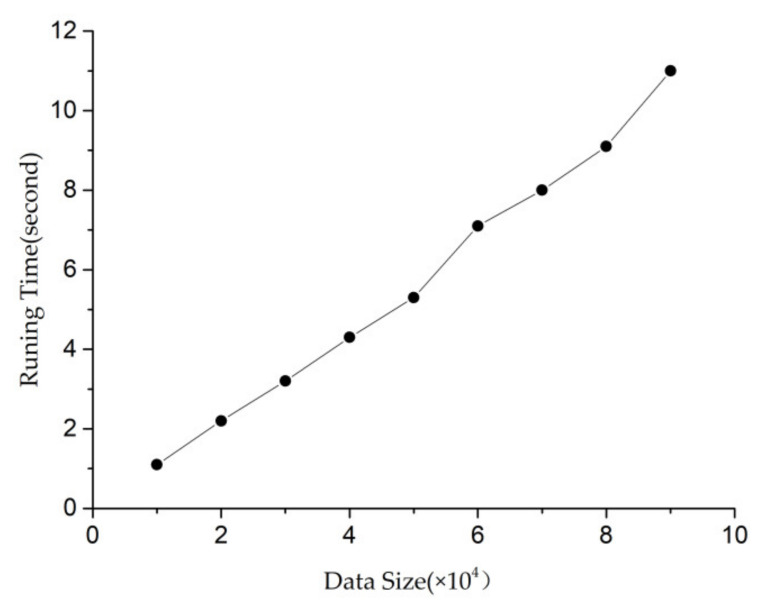
Relationship between “data scale” and “running time”.

It can be seen from the curve trend in Figure 11 that the total running time increases with the increase in data size, but it still shows a linear growth trend rather than an exponential growth. Moreover, when the data scale is 90,000, the running time to finally mine all modes is only 11 s. Therefore, the curve trend in Figure 11 can prove the efficiency of the TW-Apriori algorithm proposed in this paper, where minwin = 5000, minsup = 0.035, minconf = 0.04, mintcr = 0.1.

#### 4.1.2. Analysis of FSITW Mining Results

**Figure 12:**
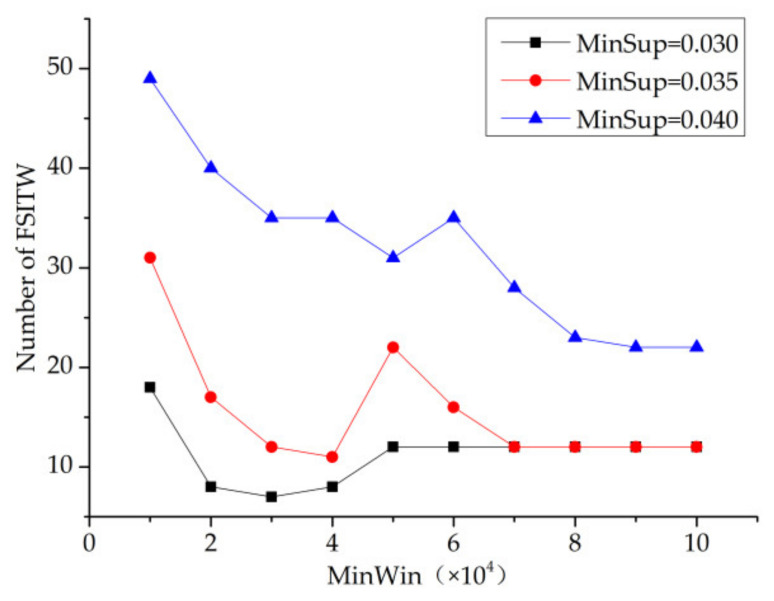
MinWin—number of FSITWs.

First, this paper will study the division of time window so as to mine the optimal minwin, as shown in Figure 12. By analyzing the three curves with minsup values of 0.030, 0.035 and 0.040, it was found that when minsup = 0.030, the number of FSITW decreased with the increase in minwin threshold. For the other two curves, it was found that the number of FSITW also showed a decreasing trend, but when the value of minwin was greater than 70,000, the two curves basically coincided; that is, when the value of minsup was 0.035 and 0.040, the impact on the number of FSITWs was the same.

Minwin = 100,000 represents that the size of the minimum time window is the total time length of the whole time series database. Moreover, from the curve of minsup = 0.030, we can see that the number of FSITWs finally mined was greater than the number of FSIs.

#### 4.1.3. Analysis of SSPTW Mining Results

In the FSITW mining stage, we found that when minsup = 0.035, the number of FSITWs excavated is the largest. Next, this paper will study the value of minimum confidence because the number of SSPTWs finally mined has a very important relationship with the values of minsup and minconf.

**Figure 13:**
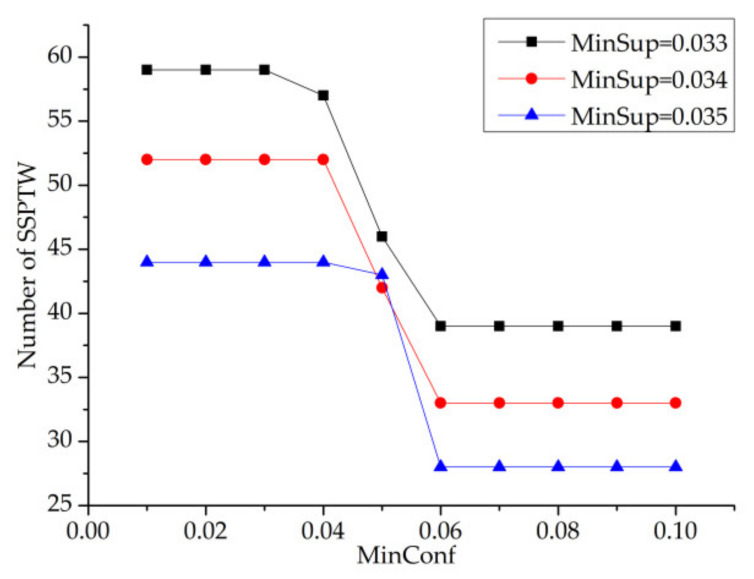
MinConf–MinSup—number of SSPTWs.

In Figure 13, minsup has three values, which are 0.033, 0.034 and 0.035, respectively. It can be seen from Figure 13 that with the increase in the minsup threshold, the number of SSPTWs finally mined gradually decreases. Moreover, when the minsup threshold is constant, the number of SSPTWs decreases with the increase in minconf value, but the number of SSPTWs does not change in [0.01,0.4] and [0.06,0.1], which indicates that the confidence has little effect on the number of SSPTWs when it is taken in these two intervals. Finally, this paper selects the representative points of minconf value, which are 0.02, 0.05 and 0.10.

**Figure 14:**
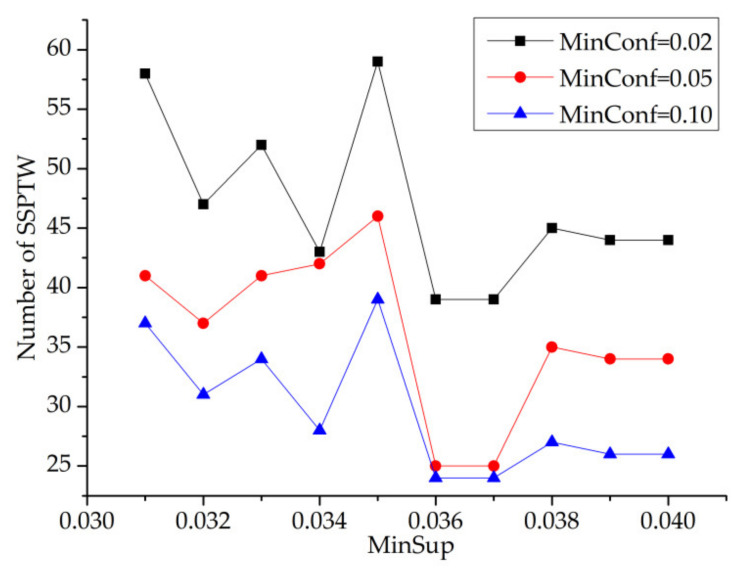
MinSup–MinConf—number of SSPTWs.

**Figure 15:**
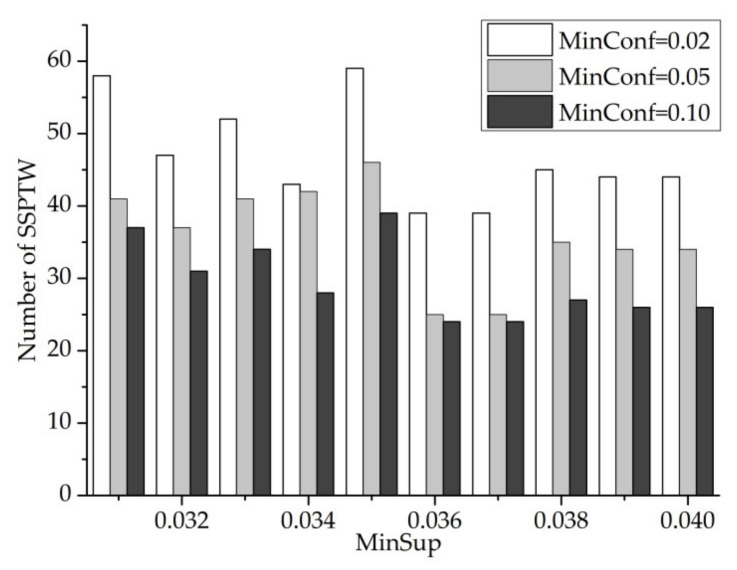
MinSup–MinConf—number of SSPTWs.

In Figure 14 and Figure 15, the values of minconf are 0.02, 0.05 and 0.10, respectively (minwin = 5000 and mintcr = 0.1). From these two figures, we can see that when minsup is constant, the number of SSPTWs decreases with the increase in minconf. However, when minconf is constant, the number of SSPTWs fluctuates greatly. It can be seen from the figure that when minsup = 0.035, the number of SSPTWs finally mined by the three curves is the largest. Therefore, when minsup = 0.035, the number of SSPTWs is greater.

#### 4.1.4. Analysis of Results of Strong SSPTW Mining

When the coverage threshold is considered, strong SSPTWs can be mined by this method. In this paper, minwin = 5000; mintcr = 0.1; and minsup = 0.33, 0.034 and 0.035, respectively.

**Figure 16:**
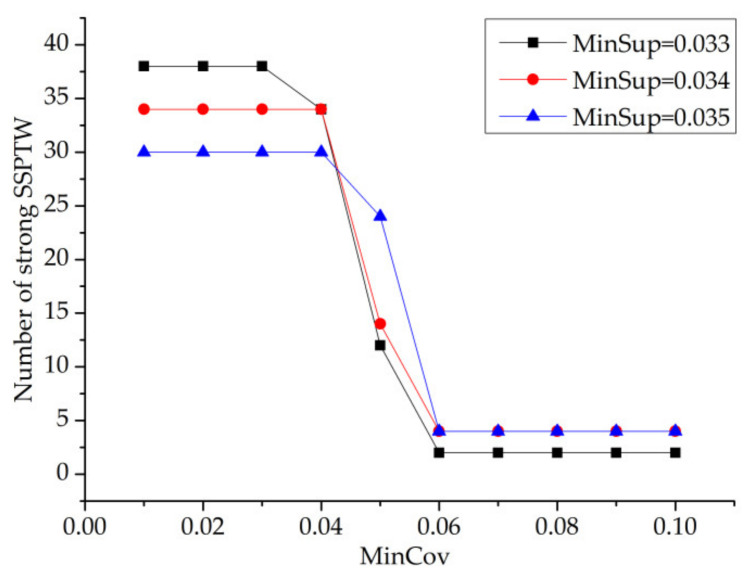
MinCov–MinSup—number of strong SSPTWs.

It can be seen from Figure 16 that, when minsup is constant, the number of strong SSPTWs finally mined decreases with the increase in the mincov value, but when mincov is in the interval of [0.01,0.4] and [0.06,0.1], the number curve of strong SSPTWs is a horizontal straight line, which indicates that the change in the mincov value in the interval of [0.01,0.04] and [0.06,0.1] has no effect on the number of strong SSPTWs.

#### 4.1.5. Analysis of the Impact of the Minimum Time Coverage Threshold on the Number of SSPTWs

This paper studies the value of mintcr and observes the influence of its value on the number of SSPTWs (minwin = 5000, minsup = 0.035, minconf = 0.04).

**Figure 17:**
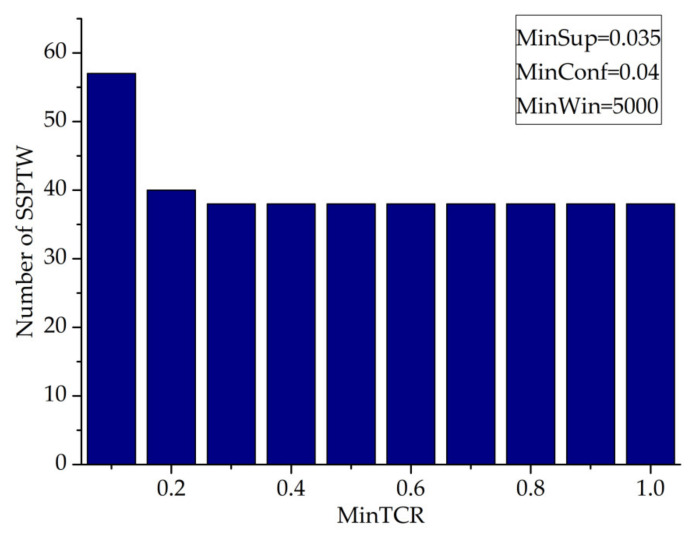
MinTCR—number of SSPTWs.

It can be seen from Figure 17 that the number of SSPTWs decreases with the increase in the mintcr value, but when the mintcr value is in the range of [0.03, 1.0], it has little effect on the number of SSPTWs.

**Figure 18:**
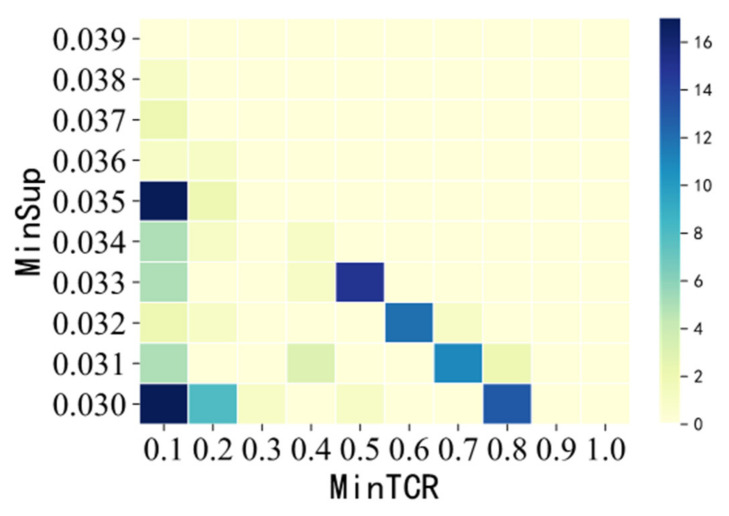
MinTCR–MinSup—number of SSPTWs.

In Figure 18, we can see the number of SSPTWs in all combinations of mincov–minsup intuitively. In addition, according to the different colors and the depth of colors in the graph, the optimal combination of mincov–minsup is obtained, which is divided into (0.1,0.03) and (0.1,0.035).

#### 4.1.6. Factor set of FSITW Mining Result Analysis

When considering the ratio constraint value of the factor set, this paper can mine the major factor set of FSITWs (minwin = 5000, minsup = 0.035, minconf = 0.1).

**Figure 19:**
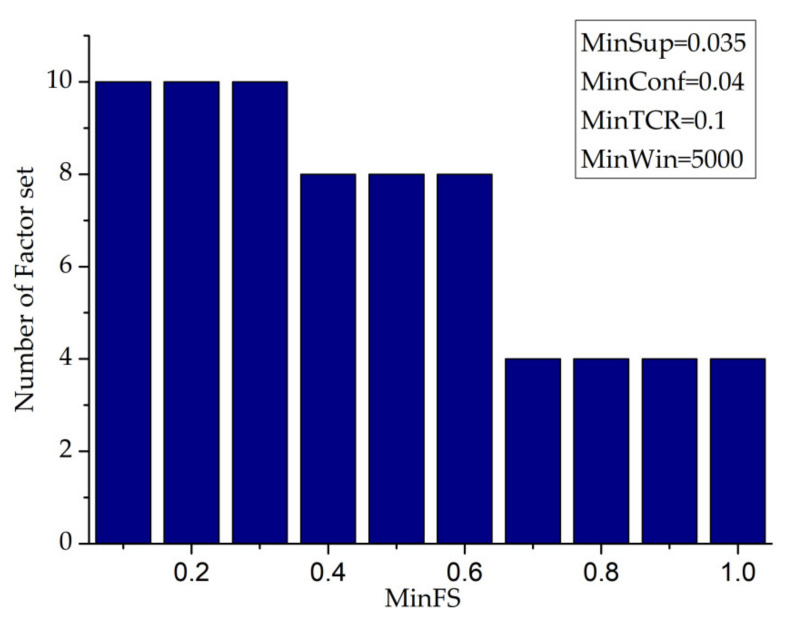
MinFS—number of factor sets.

From Figure 19, it can be seen that with the increase in the minfs value, the number of factor sets eventually mined will gradually decrease, and when the minfs value is in three intervals, [0.1,0.3], [0.4,0.6], [0.7,1.0], the change in the corresponding minfs value has no effect on the number of factor sets.

#### 4.1.7. The Periodicity Analysis of Pattern

By analyzing the periodicity of SSPTWs, we can find the periodic status set sequence patterns which satisfy the cycle width and cycle interval.

**Figure 20:**
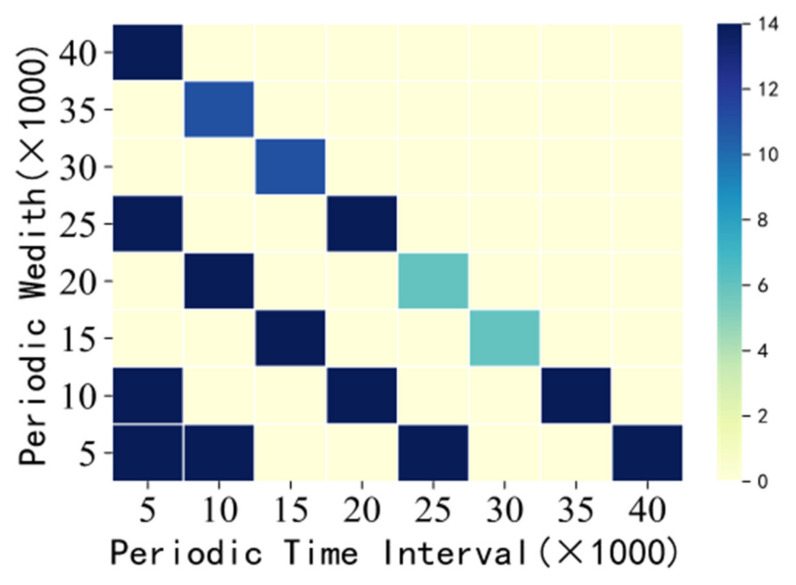
Periodic time interval–periodic width—number of periodic SSPs.

Figure 20 shows the number of periodic sequence patterns corresponding to all combinations of “period interval–period width”. Moreover, through the different colors and the changes of color depth, we can intuitively see which combinations ultimately produce the most periodic pattern data so as to provide users with valuable information and facilitate users to make decisions (minwin = 5000, minsup = 0.035, minconf = 0.04, mintcr = 0.1, minptcr = 0.1).

**Figure 21:**
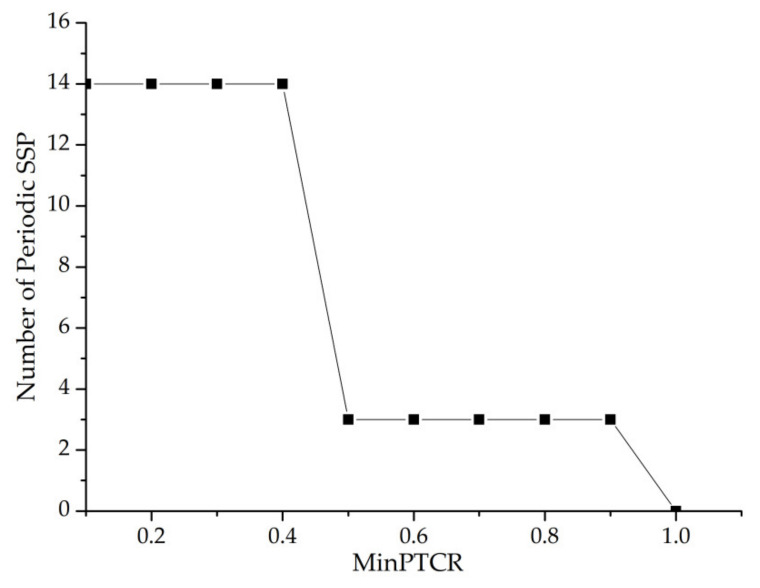
MinPTCR—number of periodic SSPs.

Next, we will fix the value of “cycle width–cycle interval” to study the periodic time coverage and observe the influence of the value of periodic time coverage on the number of periodic sequence patterns. The cycle width is 5000, and the cycle interval is 5000. From Figure 21, we can see that the number of periodic sequence patterns decreases with the increase in minptcr value. However, when the minptcr value is in the range of [0.1,0.4] and [0.5,0.9], the corresponding minptcr value has little effect on the number of periodic sequence patterns. Additionally, as the minptcr threshold increases, the regularity of the periodic SSPs becomes stronger, and the practical application value becomes greater.

### 4.2. Comparison and Analysis of Solution Results of SSPM and SSPMTW

#### 4.2.1. Comparative Analysis of FSITW Mining and FSI Mining

**Figure 22:**
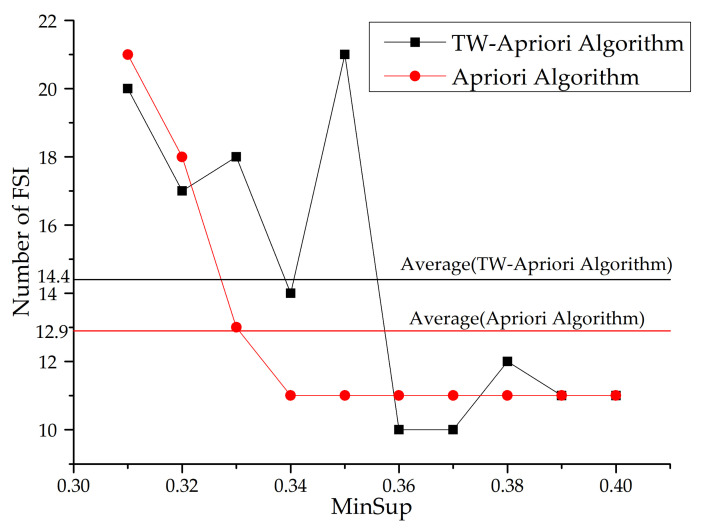
MinSup—number of FSIs.

**Figure 23:**
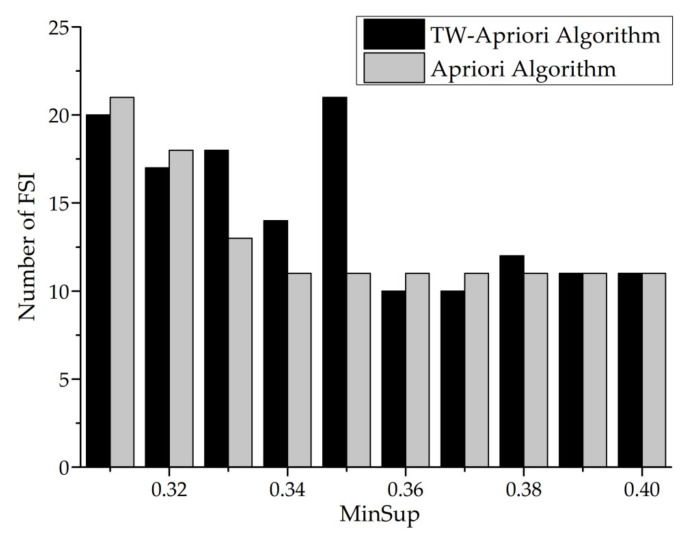
MinSup—number of FSIs.

Next, this paper will use the TW-Apriori algorithm and Apriori algorithm to mine FSI and make a comparative analysis (minwin = 5000, mintcr = 0.1). The specific figures are shown in Figure 22 and Figure 23.

It can be seen from Figure 22 and Figure 23 that the number of FSI mined by Apriori algorithm decreases with the increase of the minsup threshold, while the mining results of the TW-Apriori algorithm do not show a monotonic decreasing trend, among them; when minsup is greater than 0.35, the number of FSI decreases rapidly; while minsup is greater than 0.37, the number of FSI increases by a small margin. At this time, the number of FSI mined by Apriori algorithm does not change with the growth of minsup. When the value of minsup is in the interval [0.032,0.036] and [0.037,0.039], the number of FSIs excavated has three peaks, and in these two intervals, the number of FSIs mined by the TW-Apriori algorithm is more than that mined by traditional algorithms, especially when minsup = 0.035. The TW-Apriori algorithm mined the largest number of FSIs. In addition, it can be seen from Figure 22 that the average value of the mining results of the TW-Apriori algorithm is higher than that of traditional algorithms. In this graph, it is proved that the status set sequential pattern mining method with time window can mine the status itemset in the local time window, which makes up for the defects of traditional sequential pattern mining methods and verifies the important value and significance of SSPMTW.

#### 4.2.2. Comparison and Analysis of SSPTW Mining and SSP Mining

Table 9 shows that the TW-Apriori algorithm and the Apriori algorithm are used to mine status set sequential patterns, respectively. From the last two columns of Table 9, it can be seen that the number of SSPs mined by the two methods shows a downward trend with the increase in the support threshold. When the support threshold is 3.0%, the number of SSPs mined by the two methods is the largest, and the number of SSPs mined by SSPMTW is 40. The number of SSPs mined by traditional methods is 31; when the support threshold is the same, the number of SSPs with time windows is more than that without time windows. The average number of SSPs with time windows is 12.7, while the average number of SSPs without time windows is 5.1. The red words in the table indicate that when the minsup value is in the interval [3.4%, 3.9%] and the mintcr value is in the interval [10%, 30%] the traditional method was unable to mine SSPs, while SSPMTW can still mine a certain number of SSPs, which means that the traditional method ignores the sequence patterns existing in the local time window, and the SSPMTW proposed in this paper can solve this problem well.

Therefore, through comparative analysis, it was found that because SSPM is carried out over the whole time period of the time series database, when the support threshold increases, it may not be able to mine SSPs that meet the constraints; however, SSPMTW is carried out over the local time window, so it can mine some SSPs that do not meet the constraints in the whole time period but do meet the constraints in the local time window.

#### 4.2.3. Analysis of the Influence of Time Window and Non-Time Window on Pattern Diversity

SSPM without considering time windows can mine FSIs, SSPs, strong SSPs and factor sets of FSIs, while SSPMTW can not only mine FSITWs, SSPTWs, strong SSPTWs and factor sets of FSITWs but can also mine periodic SSPs.

Therefore, compared with SSPM, SSPMTW can mine the rules existing in the local time window and analyze the periodicity of the rules.

## 5. Discussion and Conclusions

### 5.1. Discussion

Experiments show that the proposed method can discover the relationship between items with different attributes. The algorithm proposed in this paper can mine more frequent itemsets and sequential patterns than the traditional algorithm, which shows that the traditional sequential pattern mining methods ignore the rules in the local time window, and the method proposed in this paper makes up for this. In addition, by adding the confidence threshold, coverage threshold and factor set ratio, and analyzing the periodicity of the status set sequence pattern with time windows, this paper has revealed the strong status set sequence pattern with time windows, the main factor set of frequent status itemsets with time windows and the periodic status set sequence patterns; these rules are more regular and valuable, overcoming the limitations of traditional sequential pattern mining algorithms. These more regular sequential patterns can better predict the future state of the system, make the operation of the system more stable and orderly, and reduce the entropy of the system.

The mining problem of the rule of “heatstroke→complication” mentioned above can be solved well by the method proposed in this paper. The occurrence of heatstroke and its complications is not frequent over the whole period of time. After dividing the whole study period into months, we found that the occurrence of these diseases was frequent in the months with higher temperatures. On this basis, the corresponding laws can be easily mined out. According to these rules, appropriate preventive measures can be taken for these diseases. Therefore, the method and algorithm proposed in this paper can solve some practical problems well and have certain practical significance.

### 5.2. Conclusions

In this paper, the problem of mining sequential patterns of status sets with time windows was proposed by considering the case of time windows. Firstly, the concepts involved in SSPMTW process were defined, and the corresponding properties were proposed, including some new constraints, such as minimum time window, minimum time coverage and minimum periodic time coverage. The periodicity of SSPMTWs was analyzed, and the constraints of cycle width and interval are proposed. According to the research content, this paper proposes the TW-Apriori algorithm and explains the idea of the algorithm. Finally, through small-scale and large-scale examples, the feasibility, effectiveness and efficiency of the proposed method and algorithm were verified, and a variety of patterns and rules were finally mined, such as FSITW, SSPTW, strong SSPTW, factor set of FSITW and periodic SSP; these rules can make the system more orderly and reduce the entropy of the system. Through the comparative analysis of SSPM and SSPMTW, it can be seen that compared with SSPM, SSPMTW can excavate the laws existing in the local time window and analyze the periodicity of the laws, which solves the problem of SSPM ignoring the laws existing in the local time window. In addition, by increasing the confidence threshold, coverage threshold and factor set ratio, we discovered more regular and valuable sequential patterns in the local time window, which overcomes the limitations of traditional sequential pattern mining methods.

## Figures and Tables

**Table 1:** Symbol definitions for SSPMTW models.

Symbol	Definition
*I*	A collection of all status items, symbolized as *I* = {*i*_1_, *i*_2_, …, *i_m_*}
*X*	A set of status items, such as *X*, can be represented as *X⊆I*
*D*	Collection of all events in a time series database
*T*	The entire time period in which the time series database is located
*W*	Collection of time windows
*w*	A time window, such as *w* = [*t_s_*,*t_e_*], represents a continuous time interval that starts at *t_s_* and ends at *t_e_*
*|w|*	Width of time window
*D^w^*	Collection of events occurring in the *w* time period
*|D^w^|*	Number of events occurring in the *w* time period
*D(X)^w^*	Collection of events occurring in the *w* time period that contain status itemset *X*
*|D(X)^w^|*	Number of events in *D(X)^w^*
*X*_1_ *→^w^X_2_*	Sequence *X_1_→X_2_* that appears in the *w* time window
*|X*_1_ *→X*_2_ *|^w^*	Number of occurrences of sequence *X_1_→X_2_* in the *w* time window
*sup*(*X_1_→^w^X_2_*)	Support for *X_1_→^w^X_2_* within the *w* time window can be expressed as *|X_1_→X_2_|^w^/|D^w^|*
*c*(*X*_1_*→^w^X_2_*)	Confidence for *X_1_→^w^X_2_* within the *w* time window can be expressed as *|X_1_→X_2_|^w^/|D(X_1_)^w^|*
*s*%	User-specified minimal support, *minsup*
*c*%	User-specified minimal confidence, *minconf*
ω	User-specified minimal width of time window, *minwin*
*g*%	User-specified minimal time coverage rate, *mintcr*
*d*%	User-specified minimal coverage rate, *mincov*
*u*%	User-specified minimal factor set rate, *minfs*
*e*%	User-specified minimal periodic time coverage rate, *minptcr*

**Table 2:** Temporal database.

TID	Status Itemset	TID	Status Itemset	TID	Status Itemset
1	(i_1_,1), (i_2_,2), (i_3_,2)	10	(i_1_,2), (i_2_,2)	19	(i_2_,1), (i_3_,2)
2	(i_1_,2), (i_2_,1), (i_3_,1)	11	(i_1_,1), (i_3_,2)	20	(i_2_,1)
3	(i_1_,1), (i_2_,2), (i_3_,2)	12	(i_1_,2), (i_2_,1), (i_3_,1)	21	(i_1_,1), (i_3_,2)
4	(i_1_,2), (i_2_,1), (i_3_,1)	13	(i_1_,1), (i_2_,2)	22	(i_1_,2), (i_2_,1), (i_3_,1)
5	(i_1_,1), (i_2_,2)	14	(i_1_,2), (i_2_,1), (i_3_,1)	23	(i_1_,1), (i_2_,2)
6	(i_1_,2), (i_2_,1)	15	(i_2_,2)	24	(i_1_,2), (i_2_,1), (i_3_,1)
7	(i_3_,2)	16	(i_1_,2), (i_2_,2)	25	(i_2_,2)
8	(i_1_,2), (i_3_,2)	17	(i_1_,1), (i_3_,2)		
9	(i_1_,1), (i_3_,2)	18	(i_1_,2), (i_3_,1)		

**Table 3:** Temporal database (0–1).

TID			Status Item				TID			Status Item			
	(i_1_,1)	(i_1_,2)	(i_2_,1)	(i_2_,2)	(i_3_,1)	(i_3_,2)		(i_1_,1)	(i_1_,2)	(i_2_,1)	(i_2_,2)	(i_3_,1)	(i_3_,2)
1	1	0	0	1	0	1	14	0	1	1	0	1	0
2	0	1	1	0	1	0	15	0	0	0	1	0	0
3	1	0	0	1	0	1	16	0	1	0	1	0	0
4	0	1	1	0	1	0	17	1	0	0	0	0	1
5	1	0	0	1	0	0	18	0	1	0	0	1	0
6	0	1	1	0	0	0	19	0	0	1	0	0	1
7	0	0	0	0	0	1	20	0	1	0	0	0	0
8	0	1	0	0	0	1	21	1	0	0	0	0	1
9	1	0	0	0	0	1	22	0	1	1	0	1	0
10	0	1	0	1	0	0	23	1	0	0	1	0	0
11	1	0	0	0	0	1	24	0	1	1	0	1	0
12	0	1	1	0	1	0	25	0	0	0	1	0	0
13	1	0	0	1	0	0	Sum	9	12	8	9	7	9

**Table 4:** Comparison between FSITW and FSI.

		FSITW			FSI
	Status Itemset	Time Window	Support	Time Coverage Rate	Status Itemset
L_1_	(i_1_,1)	[1–3,5]	[0.4,0.4]	0.8	(i_1_,2)
	(i_1_,2)	[1–5]	[0.48]	1.0	
	(i_2_,1)	[1,3,5]	[0.4,0.4,0.4]	0.6	
	(i_2_,2)	[1–3,5]	[0.4,0.4]	0.8	
					
	(i_3_,1)	[1,3,5]	[0.4,0.4,0.4]	0.6	
	(i_3_,2)	[1–4]	[0.4]	0.8	
L_2_	(i_1_,2)(i_2_,1)	[1,3,5]	[0.4,0.4,0.4]	0.6	∅
	(i_1_,2)(i_3_,1)	[1,3,5]	[0.4,0.4,0.4]	0.6	
	(i_2_,1)(i_3_,1)	[1,3,5]	[0.4,0.4,0.4]	0.6	
L_3_	(i_1_,2) (i_2_,1)(i_3_,1)	[1,3,5]	[0.4,0.4,0.4]	0.6	∅
Number		10			1

**Table 5:** Status-set sequential pattern with time window.

	SSPTW	TW	Support	Confidence	Coverage Rate	TCR
L1	(i_1_,1)…(i_1_,2)(i_2_,1)(i_3_,1)	※	※	※	※	※
L_2_	(i_1_,1)→(i_1_,2)	[1,3,5]	[0.4,0.4,0.4]	[0.67,1.0,1.0]	[1.0,1.0,1.0]	0.6
	(i_1_,1)→(i_2_,1)	[1,3,5]	[0.4,0.4,0.4]	[0.67,1.0,1.0]	[1.0,1.0,1.0]	0.6
	...	...	...	...	...	...
	(i_2_,1)(i_3_,1)→(i_2_,2)	[1,3,5]	[0.4,0.4,0.4]	[1.0,1.0,1.0]	[0.67,1.0,1.0]	0.6
	(i_1_,2)(i_2_,1)(i_3_,1)→(i_2_,2)	[1,3,5]	[0.4,0.4,0.4]	[1.0,1.0,1.0]	[0.67,1.0,1.0]	0.6
L_3_	(i_1_,1)→(i_1_,2)→(i_2_,2)	[1,3,5]	[0.4,0.4,0.4]	[1.0,1.0,1.0]	[0.67,1.0,1.0]	0.6
	(i_1_,1)→(i_2_,1)→(i_2_,2)	[1,3,5]	[0.4,0.4,0.4]	[0.67,1.0,1.0]	[0.67,1.0,1.0]	0.6
	...	...	...	...	...	...
	(i_1_,1)→(i_2_,1)(i_3_,1)→(i_2_,2)	[1,3,5]	[0.4,0.4,0.4]	[0.67,1.0,1.0]	[0.67,1.0,1.0]	0.6
	(i_1_,1)→(i_1_,2)(i_2_,1)(i_3_,1)→(i_2_,2)	[1,3,5]	[0.4,0.4,0.4]	[0.67,1.0,1.0]	[0.67,1.0,1.0]	0.6

**Table 6:** Major factor set of FSITW.

FSI	L_k_	Major Factor Set	TW	Support	Confidence	TCR
(i_2_,2)- [1–3,5]	L_1_	(i_1_,2)	[1,3,5]	[0.4,0.4,0.4]	[1.0,1.0,1.0]	0.6
		(i_2_,1)	[1,3,5]	[0.4,0.4,0.4]	[1.0,1.0,1.0]	0.6
		...	...	...	...	...
		(i_1_,2)(i_2_,1)(i_3_,1)	[1,3,5]	[0.4,0.4,0.4]	[1.0,1.0,1.0]	0.6
	L_2_	(i_1_,1)→(i_1_,2)	[1,3,5]	[0.4,0.4,0.4]	[0.67,1.0,1.0]	0.6
		(i_1_,1)→(i_2_,1)	[1,3,5]	[0.4,0.4,0.4]	[0.67,1.0,1.0]	0.6
		...	...	...	...	...
		(i_1_,1)→(i_1_,2)(i_2_,1)(i_3_,1)	[1,3,5]	[0.4,0.4,0.4]	[0.67,1.0,1.0]	0.6

**Table 7:** Periodic SSPs.

TW	L_k_	Periodic SSP	Support	Confidence	Coverage Rate	TCR
[1,3,5]	L_2_	(i_1_,2)→(i_2_,2)	[0.4,0.4,0.4]	[1.0,1.0,1.0]	[1.0,1.0,1.0]	0.6
		(i_2_,1)→(i_2_,2)	[0.4,0.4,0.4]	[1.0,1.0,1.0]	[0.67,1.0,1.0]	0.6
		...	...	...	...	...
		(i_1_,1)→(i_1_,2)(i_2_,1)(i_3_,1)	[0.4,0.4,0.4]	[0.67,1.0,1.0]	[1.0,1.0,1.0]	0.6
	L_3_	(i_1_,1)→(i_2_,1)→(i_2_,2)	[0.4,0.4,0.4]	[0.67,1.0,1.0]	[0.67,1.0,1.0]	0.6
		(i_1_,1)→(i_3_,1)→(i_2_,2)	[0.4,0.4,0.4]	[0.67,1.0,1.0]	[0.67,1.0,1.0]	0.6
		...	...	...	...	...
		(i_1_,1)→(i_1_,2)(i_2_,1)(i_3_,1)→(i_2_,2)	[0.4,0.4,0.4]	[0.67,1.0,1.0]	[0.67,1.0,1.0]	0.6
[2,4]	L_2_	(i_3_,2)→(i_1_,2)	[0.4,0.4,0.4]	[1.0,0.67,1.0]	[1.0,0.67,0.67]	0.6
		(i_1_,2)→(i_3_,2)	[0.4,0.4]	[0.67,0.67]	[0.67,1.0]	0.4

**Table 8:** Dataset description.

Tid	Status Itemset
Timestamp of each transaction	The status itemset contained in each transaction

**Table 9:** Comparative analysis of the two algorithms under the constraint of “mintcr–minsup”.

Number of SSPs		MinTCR (TW-Apriori)									Total	Apriori
		10%~20%	20%~30%	30%~40%	40%~50%	50%~60%	60%~70%	70%~80%	80%~90%	90%~100%		
MinSup	3.0%	17	8	1	0	1	0	0	13	0	40	31
	3.1%	4	0	0	3	0	0	11	2	0	20	12
	3.2%	2	1	0	0	0	12	1	0	0	16	6
	3.3%	4	0	0	1	16	0	0	0	0	21	2
	3.4%	4	1	0	1	0	0	0	0	0	6	0
	3.5%	17	2	0	0	0	0	0	0	0	19	0
	3.6%	1	1	0	0	0	0	0	0	0	2	0
	3.7%	2	0	0	0	0	0	0	0	0	2	0
	3.8%	1	0	0	0	0	0	0	0	0	1	0
	3.9%	0	0	0	0	0	0	0	0	0	0	0

## Data Availability

Not applicable.
